# Optimization of sports effect evaluation technology from random forest algorithm and elastic network algorithm

**DOI:** 10.1371/journal.pone.0292557

**Published:** 2023-10-20

**Authors:** Caixia Wang

**Affiliations:** Department of Primary Education, Jiaozuo Normal College, Jiaozuo, Henan, China; Instituto Tecnológico y de Estudios Superiores de Monterrey: Tecnologico de Monterrey, MEXICO

## Abstract

This study leverages advanced data mining and machine learning techniques to delve deeper into the impact of sports activities on physical health and provide a scientific foundation for informed sports selection and health promotion. Guided by the Elastic Net algorithm, a sports performance assessment model is meticulously constructed. In contrast to the conventional Least Absolute Shrinkage and Selection Operator (Lasso) algorithm, this model seeks to elucidate the factors influencing physical health indicators due to sports activities. Additionally, the incorporation of the Random Forest algorithm facilitates a comprehensive evaluation of sports performance across distinct dimensions: wrestling-type sports, soccer-type sports, skill-based sports, and school physical education. Employing the Top-K criterion for evaluation and juxtaposing it with the high-performance Support Vector Machine (SVM) algorithm, the accuracy is scrutinized under three distinct criteria: Top-3, Top-5, and Top-10. The pivotal innovation of this study resides in the amalgamation of the Elastic Net and Random Forest algorithms, permitting a holistic contemplation of the influencing factors of diverse sports activities on physical health indicators. Through this integrated methodology, the research achieves a more precise assessment of the effects of sports activities, unveiling a range of impacts various sports have on physical health. Consequently, a more refined assessment tool for sports performance detection and health development is established. Capitalizing on the Elastic Net algorithm, this research optimizes model construction during the pivotal feature selection phase, effectively capturing the crucial influencing factors associated with different sports activities. Concurrently, the integration of the Random Forest algorithm augments the predictive prowess of the model, enabling the sports performance assessment model to comprehensively unveil the extent of impact stemming from various sports activities. This study stands as a noteworthy contribution to the arena of sports performance assessment, offering substantial insights and advancements to both sports health and research methodologies.

## Introduction

The principal objective of engaging in sports activities is to foster physical activity, enhance motor skills, and facilitate both health and psychosocial development. The affirmative outcomes of sports participation are predominantly realized through the avenue of physical exertion [1]. Sports hold a prominent position in individuals’ lives, exerting substantial influence on their physical well-being. Particularly for the younger population, involvement in sports can lead to enhanced physical fitness, fortification of determination, and heightened functional capabilities, ultimately bolstering overall resilience [2]. The realm of sports encompasses a multitude of disciplines, each yielding distinct effects [3, 4]. Consequently, the endeavor to mine sports-related data becomes imperative to meticulously assess the effects and roles that various sports play.

The choice of sports information can lead to different outcomes in the constructed model, making the accuracy and credibility of the information a prerequisite for sports research. The selection and application of data mining tools and Feature Subset Selection (FSS) algorithms are crucial [5]. Qian and Liu (2020) discovered that traditional data collection methods had limited statistical data and low comprehensive analytical capabilities, rendering them ineffective in analyzing sports training indicators. Data mining models based on deep learning optimization can address this issue and enhance the model’s effectiveness [6]. Data mining tools can offer sound guidance for sports training and contribute to the advancement of physical education and well-being [7]. FSS plays a pivotal role in data mining, reducing time in the model training process, enhancing efficiency, and improving generalization capabilities [8]. Rojas-Valverde et al. (2019) identified that employing big data mining technology to select sports features could analyze the occupational health of sports athletes, significantly contributing to enhancing physical performance and sports technology detection [9]. In the realm of sports data mining, utilizing technologies like Naive Bayes, artificial neural networks, and decision trees within machine learning to construct sports prediction models through data FSS can heighten prediction accuracy and efficiency, enabling sports outcome predictions [10]. Bunker and Thabtah (2019) observed that using neural network technology in machine learning to construct sports outcome prediction models could facilitate data classification and FSS, enhancing the accuracy of sports and competition prediction outcomes [11]. Chen et al. (2021) explored the utilization of multiple adaptive regression splines, k-nearest neighbors, extreme learning machines, extreme gradient boosting, and stochastic gradient boosting to analyze sports data. The prediction model’s accuracy, built on FSS, significantly improved, enabling accurate predictions of sports outcomes [12]. Yuan and Cao (2023) employed a range of data mining techniques to explore speech rules at an advanced descriptive level, thereby uncovering diverse disparities in dimensions such as annual consumption, degree of participation, and consumption patterns related to sports and leisure activities among professional women. This thorough analysis contributes to a comprehensive comprehension of professional women’s engagement in sports and leisure pursuits, offering valuable recommendations and strategic orientations to stimulate their active participation within these domains [13]. Komitova et al. (2023) discerned that the temporal information inherent in most sports data yields notable benefits. The incorporation of the temporal dimension of time series into data mining profoundly augments the predictive capability of sports-related information [14].

This study endeavors to explore the profound impact of sports activities on physical health by harnessing advanced data mining and machine learning techniques. It aims to furnish a scientific foundation for the judicious selection of sports and health promotion strategies, thereby fostering the propagation of wholesome lifestyles and the advancement of the sports domain. Guided by the Elastic Net algorithm, a sports performance assessment model is erected, setting itself apart by contrasting with the conventional Least Absolute Shrinkage and Selection Operator (Lasso) algorithm. The primary objective is to delve into the factors influencing sports activities vis-à-vis physical health indicators. Furthermore, the study integrates the Random Forest algorithm, yielding a comprehensive assessment of sports performance across four dimensions: wrestling-type sports, soccer-type sports, skill-based sports, and school physical education. Employing the Top-K metric as the evaluation criterion and juxtaposing it with the high-performance Support Vector Machine (SVM) algorithm, accuracy is scrutinized across three distinct criteria: Top-3, Top-5, and Top-10. The innovation inherent in this study resides in the fusion of the Elastic Net and Random Forest algorithms. This fusion facilitates a holistic consideration of the influencing factors of diverse sports activities on physical health indicators. Through this integrated approach, the research attains a heightened capacity to precisely evaluate the ramifications of sports activities. It aptly unveils the diverse impacts engendered by various sports types on physical well-being, thus bestowing a refined assessment tool for sports performance detection and health enhancement. The incorporation of the Elastic Net algorithm in model construction during the feature selection stage optimizes the process, more adeptly capturing the pivotal factors steering varied sports activities. Simultaneously, the introduction of the Random Forest algorithm amplifies the model’s predictive prowess, enabling the sports performance assessment model to comprehensively illuminate the extent of influence wielded by distinct sports activities. In summation, this study makes a substantial stride in the realm of sports performance assessment. Its offerings extend to significant insights and innovations in the arena of sports health and research practices.

## Literature review

Elastic networks and random forests find widespread application in data mining and feature selection. Klösgen (1996) elucidated information types and the characteristics of the knowledge discovery process within the realm of knowledge discovery methods in databases. The construction of statements in data mining systems relies on a pattern language, encompassing rule patterns, average patterns, and deviation patterns. While most data mining systems support a single pattern type, the computation of statement quality necessitates the utilization of facts derived from the first level of the statistical query language [15]. Wrobel (1997) emphasized the importance of identifying statistically unusual subgroups within multi-relational databases. The integration of optimistic estimation and minimum-support pruning, involving optimized refinement operators and sampling techniques, enhances the efficiency of data mining. These early investigations laid the theoretical groundwork for subsequent data mining techniques employed in sports [16]. Knobbe et al. (2017) focused on athlete-specific fitness insights for improved training optimization. They formulated promising feature sets and employed diverse techniques, including univariate linear regression and subgroup discovery, to extract relevant features. Nonlinear models surpassed linear models in performance within this context [17]. de Leeuw et al. (2022) advanced the prediction of overuse injuries among professional volleyball players during matches. Their machine learning approach incorporated training loads and health metrics, resulting in improved predictive capabilities [18]. Imbach et al. (2022) conducted a comparative study of variable dose-response models, elasticity networks, principal component regression, and random forest models. Elasticity networks exhibited superior generalization and predictive accuracy, underscoring the significance of constructing a generalization enhancement procedure for predictive modeling [19]. Pamukcu (2019) explored the hybrid smoothed covariance with an informationally complex estimator of a data-adaptive elastic net prediction model. This approach optimized the selection of mixed covariance estimators, enhancing accuracy in data mining and feature selection [20]. Yao et al. (2021) demonstrated the efficacy of the elastic net algorithm in feature selection. When coupled with the random forest algorithm, it improved prediction accuracy, particularly for genetic diseases [21]. Shi et al. (2019) combined lasso descent dimensionality reduction and random forest method for drug target action prediction. Dimensionality reduction and feature extraction improved the accuracy and precision of the model [22]. Dikananda et al. (2021) evaluated plain Bayes, decision tree, and random forest algorithms for data classification. The random forest algorithm achieved a classification accuracy of 60%, showcasing superior performance in data mining and feature selection [23]. Alfredo and Isa (2019) leveraged the Random Forest Algorithm and Extreme Gradient Boosting algorithm for soccer match history data. A prediction model for match outcomes was established. While both algorithms were employed, the Random Forest algorithm slightly outperformed the Extreme Gradient Boosting model in terms of accuracy [24]. Yildiz (2021) highlighted the potency of machine learning techniques across various scientific domains, including sports. Machine learning applications encompass predicting match outcomes, sports results, and team classification [25]. With the continued development of the Internet of Things (IoT), supplementary and exploratory data mining, along with increased access to various cloud computing platforms, accelerated the realization of planned technological innovations [26]. Chen and Ye (2023) extracted key volleyball match information using data mining and wireless communication network techniques. Real-time data retrieval enhancement was achieved through frequent record searches [27]. Mingchan (2023) established an intelligent sports platform utilizing data mining and machine learning. The platform played a pivotal role in advancing sports development [28]. Table 1 presents a comparison of various techniques utilized in previous studies.

**Table 1:** Technical comparison.

Author	Technologies
Klösgen (1996)	Pattern languages, specific patterns
Wrobel (1997)	Optimized refinement operators, sampling methods
Knobbe et al. (2017)	Resilient networks, Lasso, subgroup discovery
Pamukcu (2019)	Elastic net prediction model with hybrid smoothed covariance estimator
Shi et al. (2019)	Lasso downgrading, random forests, drug target action prediction models
Alfredo and Isa (2019)	Random Forest, Extreme Gradient Boosting
Yildiz (2021)	Machine learning, data mining, wireless communication networks
de Leeuw et al. (2022)	Machine learning, training load, health metrics
Mingchan (2023)	Data mining, machine learning, intelligent sports platforms

In summary, the application of elastic network and RF algorithms typically entails data mining and feature data selection. Utilizing these two algorithms can enhance the prediction accuracy of the model. However, the elastic network and RF algorithms are currently less commonly employed in sports research, primarily concentrating on predicting sports match outcomes and classifying sports teams. There are limited studies on the impacts of sports, and certain gaps remain.

## Materials and methods

### Data mining and traditional algorithms

**Fig 1:**
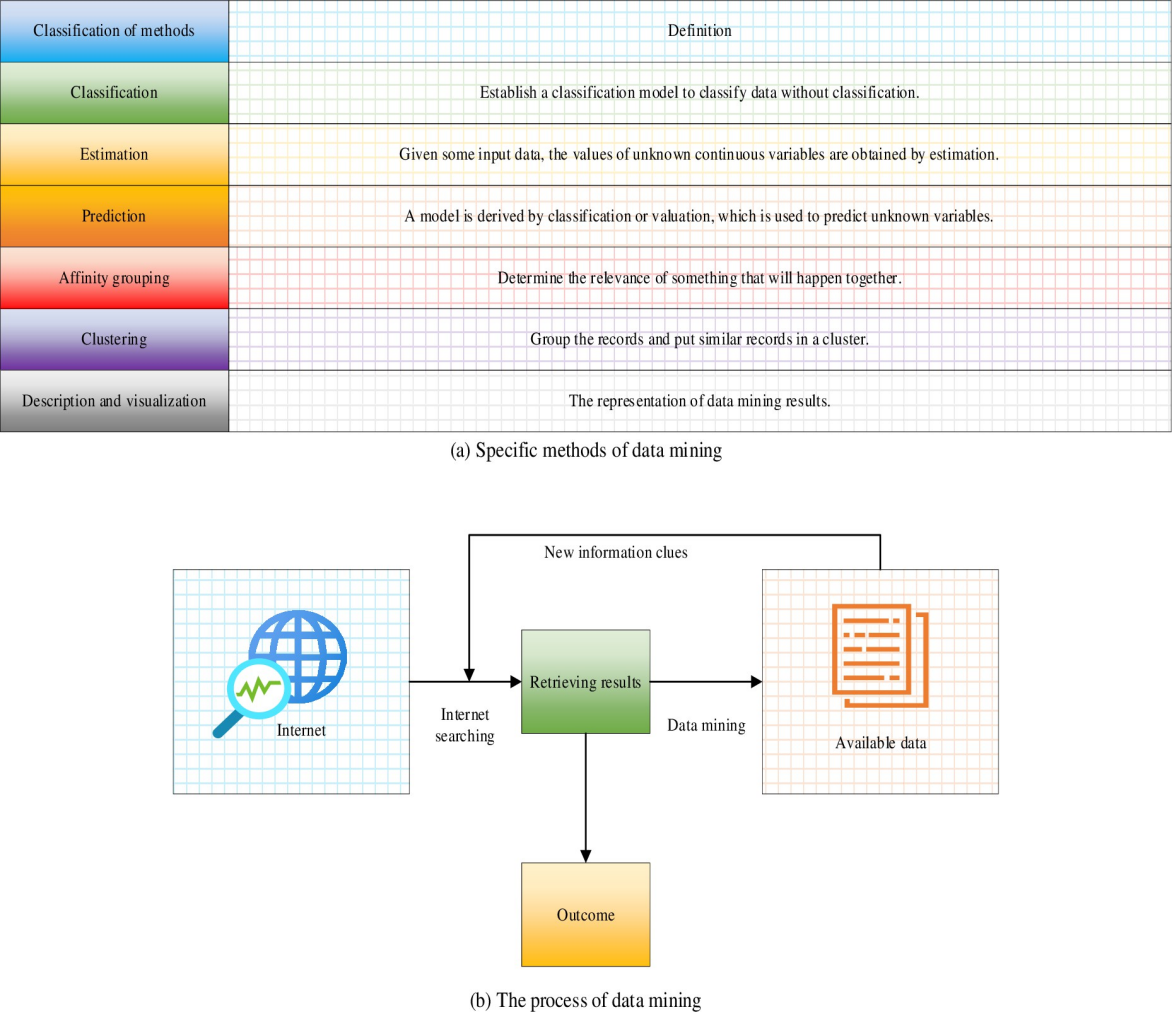
Specific methods and processes of data mining. (a) Specific methods of data mining. (b) The process of data mining.

Data mining involves the automated search within a large dataset to acquire specific, relevant, and valuable information. It is also a process of knowledge discovery [29]. Data mining generally encompasses seven stages: data cleaning, integration, selection, transformation, mining, evaluation, and representation [30]. Fig 1 displays the specific methods and flow of the data mining process.

As depicted in Fig 1, data mining involves the classification of Internet-based information, the evaluation and prediction of classified data, and the realization of correlation grouping. It employs clustering algorithms and visualizations to describe, output, and represent valid information data.

**Fig 2:**
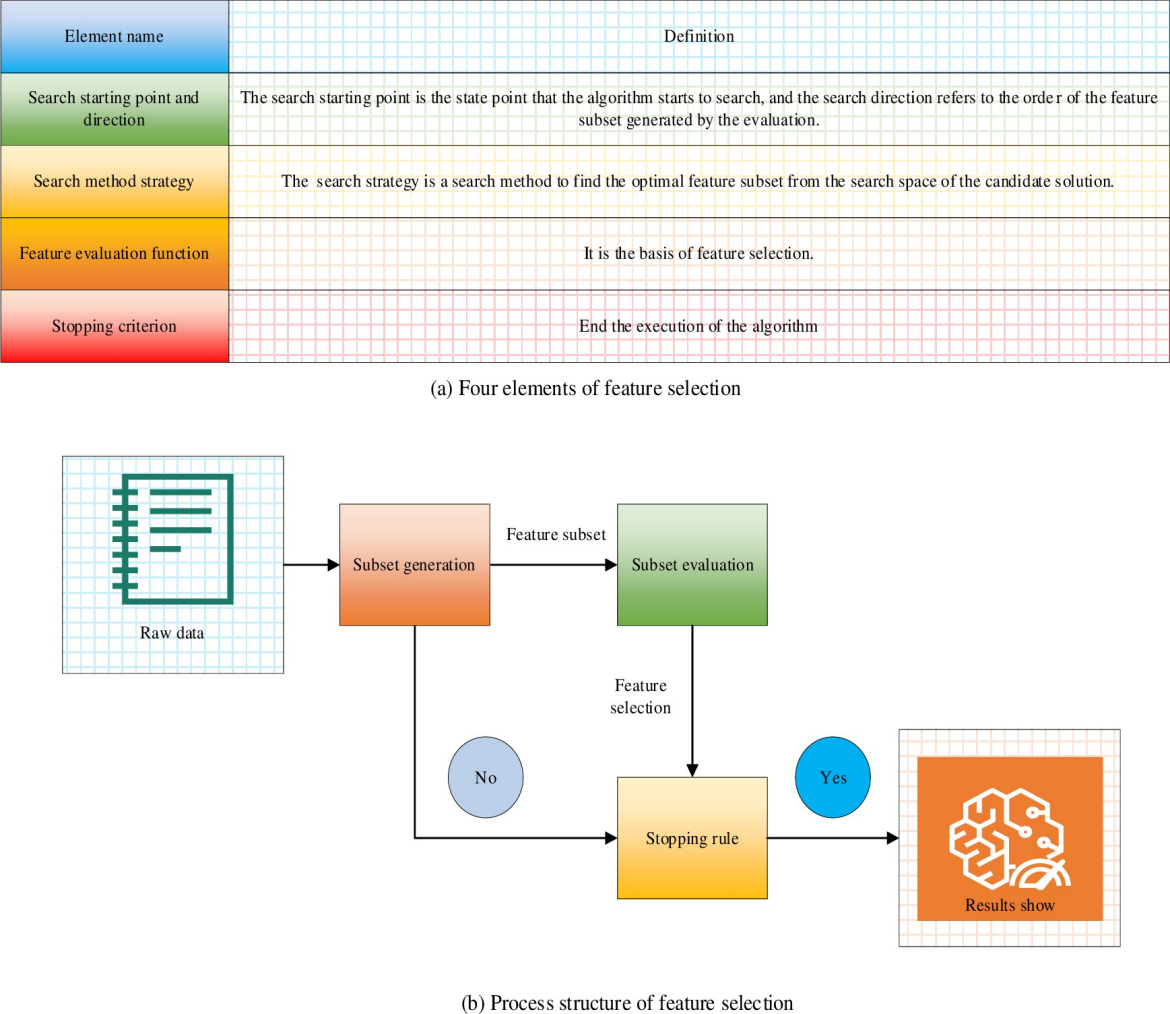
Four elements and specific process of FSS. (a) Four elements of feature selection. (b) Process structure of feature selection.

FSS, in general, refers to utilizing existing features for calculations to select crucial data that can optimize system-specific indicators [31]. FSS is divided into four components: the generation process, evaluation function, stopping criterion, and verification process [32]. The four elements of FSS and the specific process are illustrated in Fig 2.

Fig 2 suggests that the four elements of FSS are the search starting point and direction, search method strategy, feature evaluation function, and stopping criterion. It is only by relying on these four aspects that the FSS algorithm framework can be established to ensure the screening of feature data.

**Fig 3:**
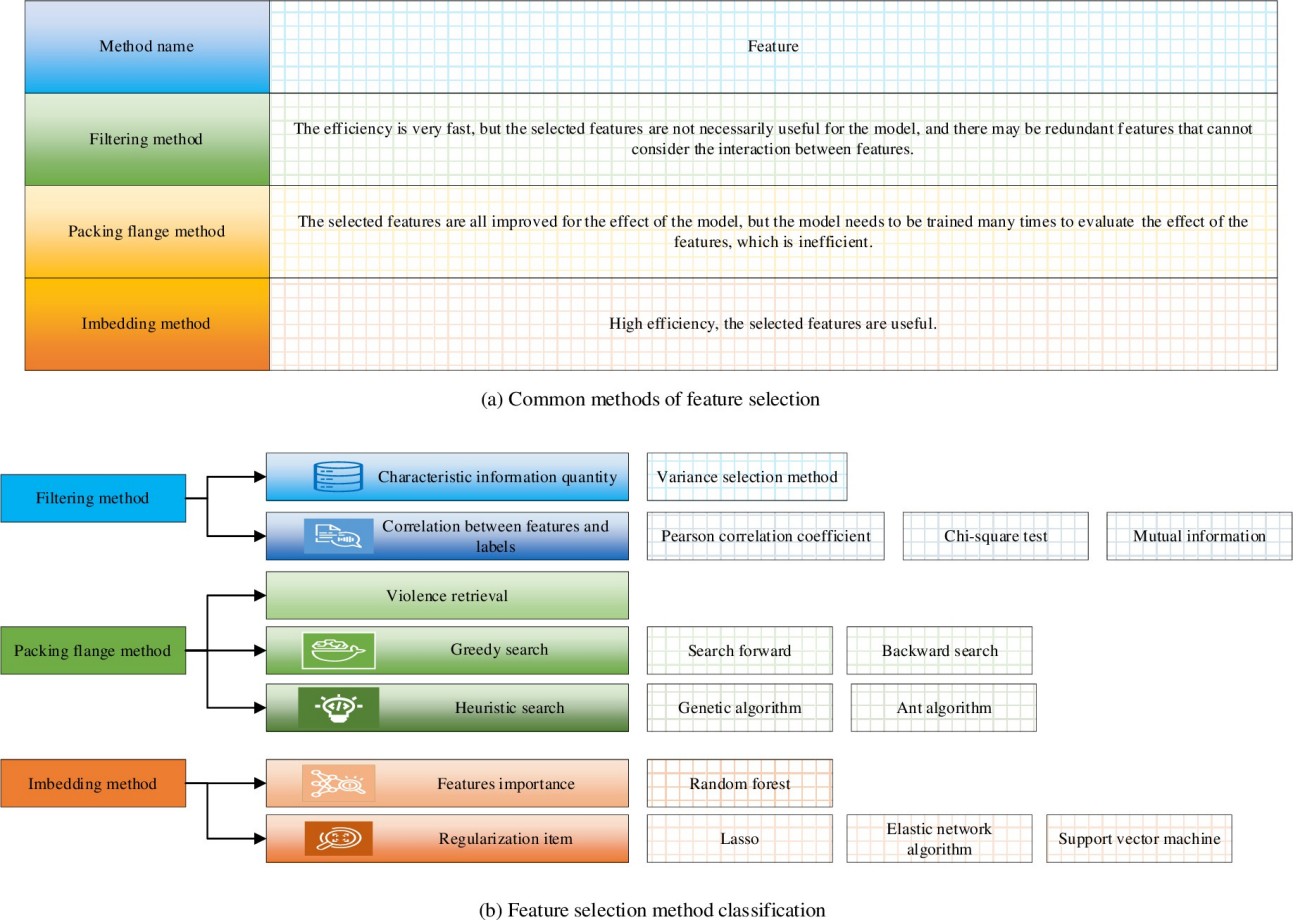
Common methods and specific classification of FSS. (a) Common methods of feature selection. (b) Feature selection method classification.

Feature selection plays a pivotal role in enhancing the efficacy of machine learning models. Notably utilized methods encompass the variance selection method, chi-square test, genetic algorithm, random forest, Lasso algorithm, support vector machine algorithm, and elastic network algorithm. Each approach meticulously sieves significant features from distinct viewpoints, bolstering the model’s generalization capability and predictive accuracy. In real-world applications, the judicious selection of appropriate feature selection methods can aptly optimize model performance, aligning with data characteristics and problem specifications. The comprehensive compilation of common methods and their specific classification is presented in Fig 3.

As depicted in Fig 3, filtering, wrapping, and embedding methods stand as commonly employed feature selection approaches that wield significance within the realm of data analysis and machine learning. These methods are instrumental in discerning the most informative features from an extensive feature pool, thereby augmenting model performance and curtailing dimensionality. Filtering, a straightforward method, finds wide application in feature selection and data preprocessing. Operating independent of specific machine learning algorithms, it sifts out features exhibiting a high correlation with the target variable. This is achieved through initial evaluation and ranking of features. Prominent filtering methods encompass Pearson’s correlation coefficient, mutual information, chi-square test, and more. Filtering methods are lauded for their computational efficiency and model-agnostic nature, yet they may overlook intricate relationships between features and the target variable. Wrapping methods enlist specific machine learning algorithms to gauge feature quality, consequently identifying the optimal subset of features. This is achieved through iterative model construction and evaluation utilizing techniques such as cross-validation. While wrapping methods tend to be time-intensive due to the need for multiple model pieces of training, they aptly capture the impact of features on model performance. Recursive feature elimination and recursive feature addition are common wrapping methods. Embedding methods merge feature selection into the model training process, allowing features to be selected concurrently with model optimization. This approach perceives feature selection as an integral component of model refinement, tuning feature weights through regularization or alternative techniques to facilitate automated feature selection. Noteworthy embedding methods include Lasso regression, ridge regression, and elastic networks. Integrating feature selection and model training, embedding methods amalgamate the merits of both filtering and wrapping techniques, rendering them a more comprehensive form of feature selection.

Tibshirani (1996) introduced the Lasso algorithm, a regression approach leveraging regularization terms for compressed estimation. Distinguished by its capacity for subset shrinkage preservation, Lasso’s algorithm adeptly addresses biased estimation in intricate covariate data scenarios, positioning it as a prominent traditional technique for feature selection [33].

SVM is a type of binary classification generalized linear classifier within supervised learning, offering advantages such as superior classification performance, high robustness, and high sparsity. It is the only machine learning algorithm that can be employed without kernel tricks and finds widespread use in recognition [34].

### Elastic network algorithm model design

**Fig 4:**
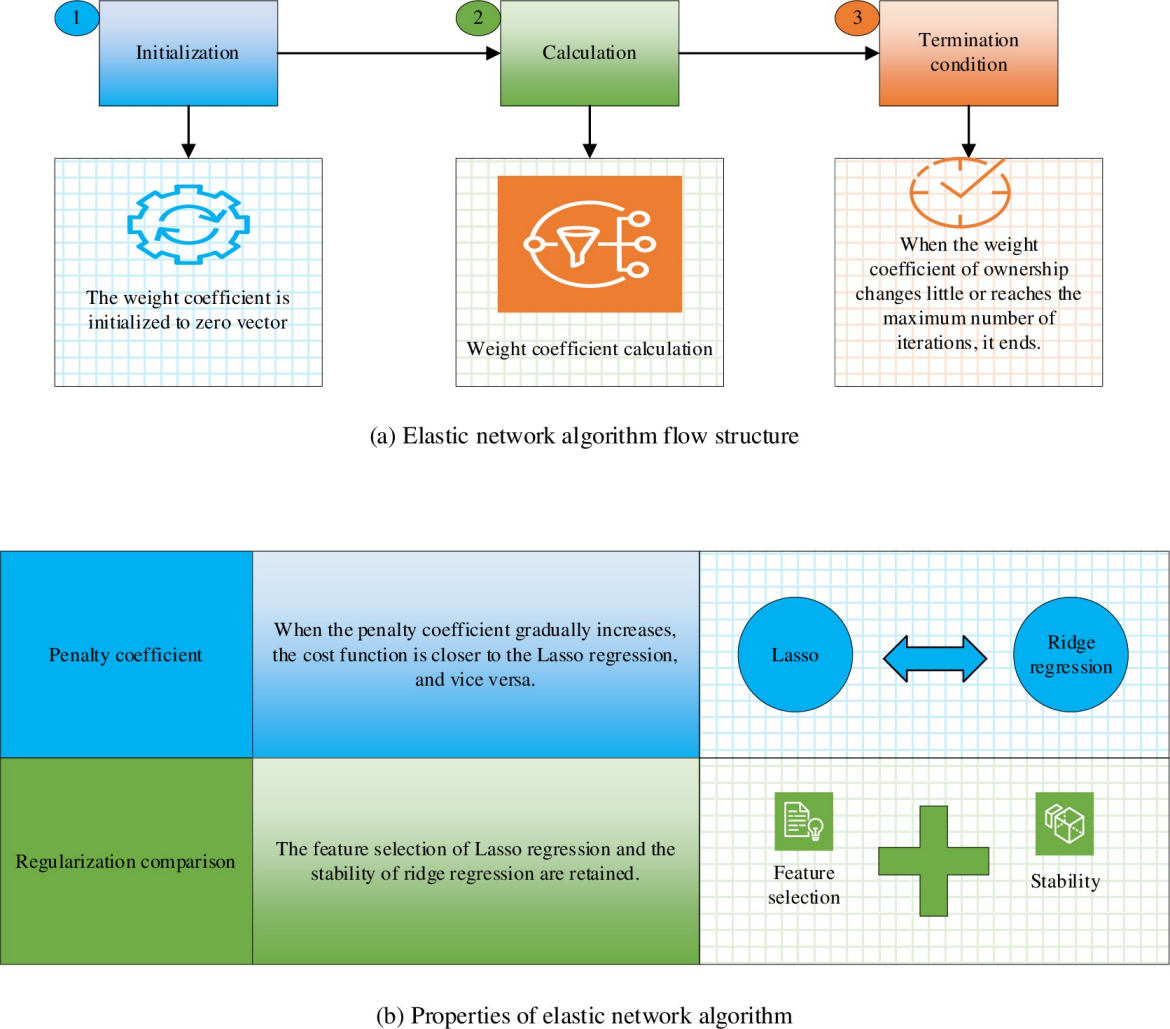
Process structure and algorithm properties of elastic network algorithm. (a) Elastic network algorithm flow structure. (b) Properties of elastic network algorithm.

An elastic network is a linear model that can utilize both L1 and L2 penalty terms in the objective function. This structure allows it to penalize the model’s complexity and simplify the overall structure [35, 36]. The cost function of the elastic network regression algorithm combines regularization techniques from both the Lasso regression and the ridge regression algorithms [37]. The process structure and algorithm properties of the elastic network algorithm are illustrated in Fig 4.

Based on Fig 4, when employing the elastic network algorithm, the weight coefficients need to be initialized initially. Next, all weight coefficients should be computed to determine the optimal solution considering only a single variable. It’s important to note that the size of the penalty coefficient is a decisive factor that dictates the bias of the elastic network cost function toward either the Lasso regression algorithm or the ridge regression algorithm.

In ***L*1** regularization, considering the parameter as ***x***_***m***_, the loss of the unregularized term is denoted as ***L***(***X***), and ***α*** serves as the hyperparameter governing regularization magnitude. The loss function is computed as Eq (1).


$$L1=L(X)+\alpha \sum_{m=1}^{n}\left|x_{m}\right|$$ (1)

In contrast, ***L2*** regularization introduces the ***L2*** norm into the loss function as a penalty term, resulting in the following loss function for ***L2***:

$$L2=L(X)+\alpha \sum_{m=1}^{n}x_{m}^{2}$$ (2)

In this context, the value of ***x***^***2***^ diminishes for ***x*** values smaller than 1.

The loss function for the composite effect of ***L1*** and ***L2*** regularization on the robust network is determined by Eq (3).


$$L=L(X)+\alpha [\alpha \sum_{m=1}^{n}\left|x_{m}\right|+\alpha \sum_{m=1}^{n}x_{m}^{2}]$$ (3)

A sports impact evaluation model is formulated using an elastic network algorithm. Let’s assume that the property set of the database is ***S***, the database dimension is ***K***, the category label of the sample is denoted as ***A***, and ***γ*** is the regression coefficient column vector in ***K*** dimensions. Thus, the model can be represented as Eq (4).


$$min{\left\|A-S\gamma \right\|}_{2}^{2}+\alpha_{1}{\left\|\gamma \right\|}_{1}+\alpha_{2}{\left\|\gamma \right\|}_{2}^{2}$$ (4)

### RF optimization model design

**Fig 5:**
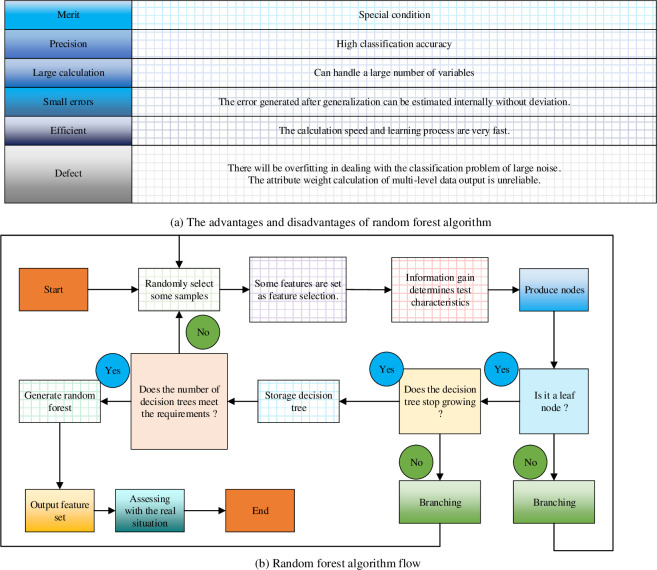
Advantages, disadvantages, and specific process of RF algorithm. (a) The advantages and disadvantages of random forest algorithm. (b) Random forest algorithm flow.

The random forest algorithm, introduced in 1995, represents a classifier comprising numerous decision trees [38]. The optimal classification outcome in the random forest algorithm is determined by the collective vote of each decision tree, with the majority of categories influencing the final output category [39, 40]. A depiction of the advantages, disadvantages, and specific processes of the random forest algorithm is presented in Fig 5.

Based on Fig 5, the RF algorithm is a highly accurate classifier that can strike a balance between errors. It possesses the capability to rapidly process extensive variable data and assess the significance of variables.

In the context of feature information, assume that ***E*** is a discrete random variable with a total of ***n*** values, and the probability of taking the ***m***-th type is denoted as ***P***. The entropy ***R***(***E***) of this random variable can be calculated using Eq (5).


$$R(E)=\sum_{m=1}^{n}P(e_{m})\log_{2}P(e_{m})$$ (5)

Feature ***T*** is provided for W classification. The information gain provided is calculated via Eq (6).


U(T)=R(T)−R(W|T) (6)

An optimization model for the RF algorithm is established. Assume the indicator feature is ***a***. The set of samples based on this indicator feature is denoted as ***H***. ***P***_***z***_ (***z*** = ***1***,***2***,..,|***f***|) represents the proportion of the ***z***-th sample in the sample set. Thus, the information entropy of the model can be defined as Eq (7).


$$R(H|=\sum_{z=1}^{\left|f\right|}P_{z}\log_{2}\;P_{z}$$ (7)

Feature ***J*** is used to divide the sample set. The resulting branch is ***φ***. The information gain is provided by Eq (8).


$$Gain(H,J)=R(H)-\sum_{\varphi =1}^{\varphi }\frac{\left|H^{\varphi }\right|}{\left|H\right|}R(H^{\varphi })$$ (8)

The Top-K metric serves as an evaluation measure utilized to assess the performance of a ranking model. It quantifies the accuracy of the model’s initial K predictions. This metric is commonly applied in diverse domains, including information retrieval, recommender systems, and natural language processing. In the Top-K metric, the parameter K signifies the position within the ranking of predictions, typically represented as a positive integer.

In this study, within the framework of the elasticity-based network sports effect model, the selection of metric attributes with substantial influence on physical metrics is achieved through the integration of the Random Forest feature selection method. This is accomplished by analyzing physical metrics data prior to and after sports activities. During algorithm implementation, distinct decision trees are constructed for various sports categories, enabling analysis of the extent to which each sport affects physical indicators. The algorithm’s accuracy is substantiated through comparison with authentic data, thus facilitating an assessment of the impact of sports activities.

The specific procedures for formulating the optimization model encompass the following steps: Commencing with the collected sports data, features germane to the research objectives are identified, serving as the foundational criteria for partitioning the decision tree. Multiple training data subsets are generated through the random selection of samples. Each of these subsets constitutes a portion of the original data. A distinct decision tree is constructed for each training data subset by employing a decision tree algorithm. The computation of the prediction error rate ensues, involving the selection of pertinent out-of-bag data. This entails the introduction of random noise interference to the features of all out-of-bag data samples, followed by the recalibration of the error assessment for the out-of-bag data. At each decision node, a subset of features from the feature set is deliberately chosen, serving as the basis for the subsequent split. Multiple autonomous decision trees amalgamate to form a random forest. Each tree corresponds to a distinct subset of autonomous samples, with each independently performing feature selection and splitting procedures.

## Experimental data design

**Fig 6:**
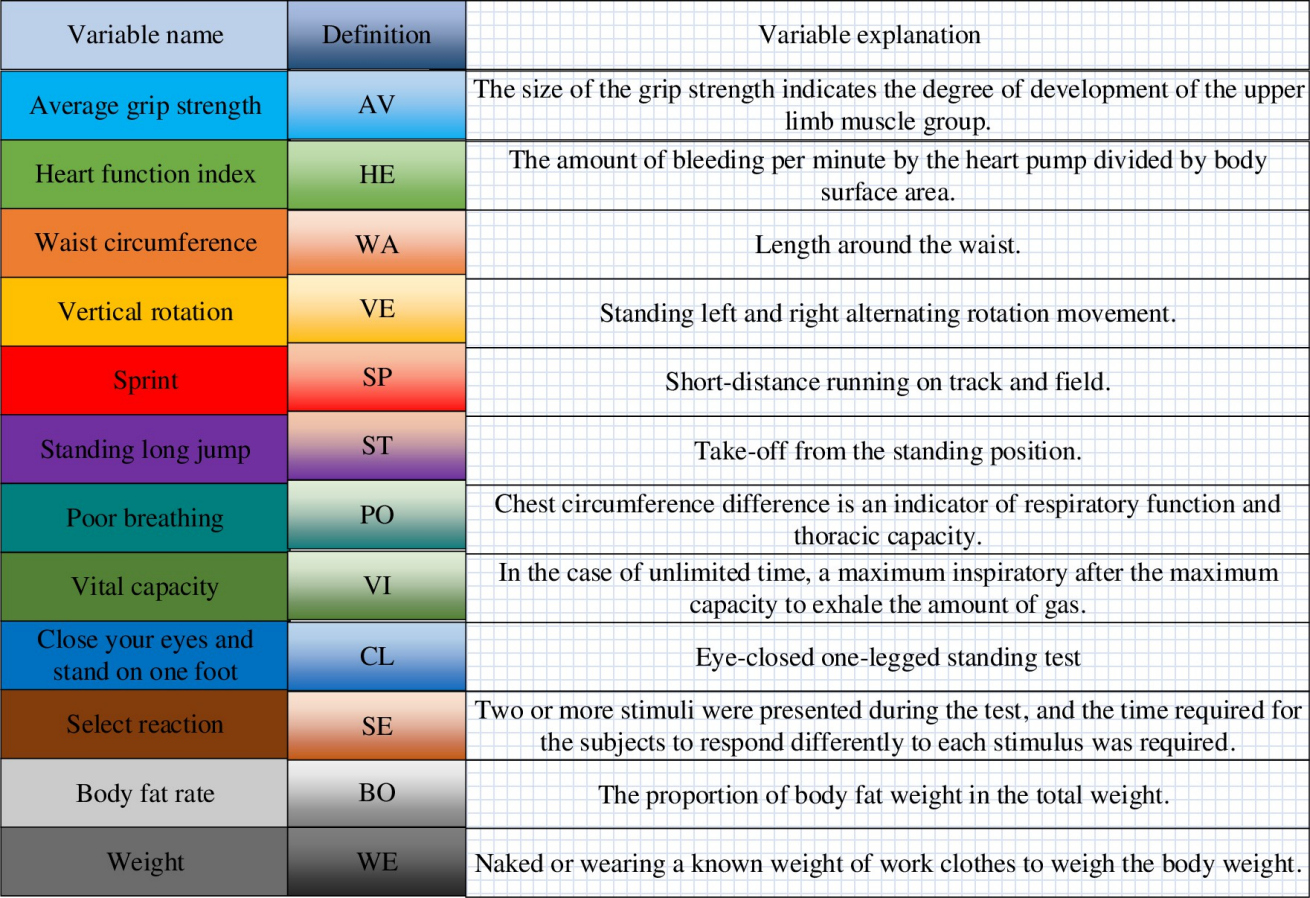
Model variable settings and variable definitions.

Here, a sports impact evaluation model is established using an elastic network algorithm, and a sports impact evaluation model is optimized with the RF algorithm. The database employed includes data from the Fifth National Physical Fitness Monitoring Bulletin and the adolescent health theme database within the national population health science data. The Fifth National Physical Fitness Monitoring Bulletin constitutes the fifth nationwide physical fitness monitoring initiative conducted by the General Administration of Sport in 2020. The monitoring targets encompass young children aged 3 to 6 years, adults aged 20 to 59 years, and elderly individuals aged 60 to 79 years. It covers 31 mainland Chinese provinces, autonomous regions, and municipalities directly under the Central Government. The method of multi-stage stratified random cluster sampling was employed. Monitoring was carried out from August to December 2020, with targets selected from 1,732 institutions, enterprises, schools, kindergartens, and administrative villages nationwide. A valid sample of 202,123 people was obtained, including 40,222 children, 121,928 adults, and 39,973 elderly individuals. The adolescent health theme database utilizes various interdisciplinary disciplines, such as kinesiology, psychology, and sociology, to provide parameters and data support for adolescents’ mental psychology, quality of life, social health, and sports behavior. Established in 2015, the adolescent health theme database engages in long-term follow-up research on adolescent health. It stands as a relatively comprehensive and extensive database of interdisciplinary index system research in China. The data selected herein comprise information on the physical condition of young people and their sports behavior spanning the years 2015 to 2020. Fig 6 provides the specific model variable settings and variable definitions.

From Fig 6, the model variables mainly include average grip strength, cardiac work index, waist circumference, standing turn, sprinting, body weight, standing long jump, poor breathing, lung capacity, closed eye monopod, selective response, and body fat rate.

## Analysis of the importance of sports impact and the accuracy of the model

### Analysis of the results of the importance of the impact of sports

**Fig 7:**
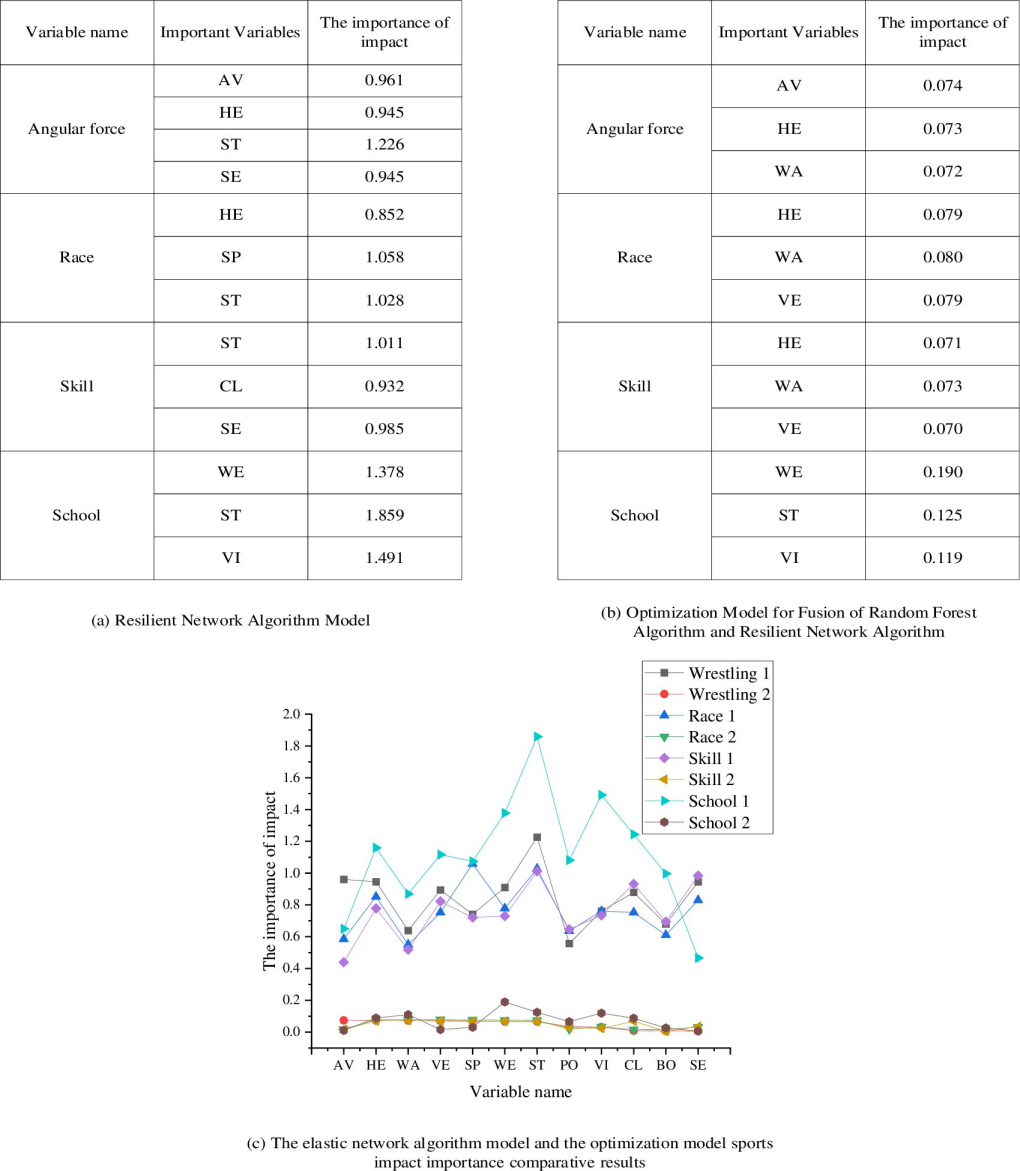
The elastic network algorithm model and the optimization model sports impact importance analysis results. (a) Resilient Network Algorithm Model. (b) Optimization Model for Fusion of Random Forest Algorithm and Resilient Network Algorithm. (c) The elastic network algorithm model and the optimization model sports impact importance comparative results.

The elastic network algorithm is employed to create a sports impact evaluation model to examine the influencing factors on physical fitness indicators in sports. The primary focus of the study is on sports as the main research variable, utilizing physical condition data and sports behavior data from young individuals spanning the years 2015 to 2020. The analysis also delves into assessing the significance of the impact of various types of sports. Fig 7 shows the elastic network algorithm and optimization model sports impact importance analysis results.

As depicted in Fig 7, the study’s outcomes reveal the significance of the impact of distinct categories of sports—cornering sports, competitive foot sports, skill sports, and school sports—analyzed through the lens of the elastic network algorithm model. For instance, in the domain of jousting sports, pivotal factors encompass the standing long jump, average grip strength, cardiac performance index, and choice reaction. These factors hold respective importance values of 1.226, 0.961, 0.945, and 0.945. In the context of footrace sports, the cardiac performance index, sprinting, and standing long jump bear substantial influence, with importance values of 0.852, 1.058, and 1.028, respectively. Similarly, skill sports exhibit the influence of factors such as standing long jump, one-legged stance, and choice reaction, carrying significance values of 1.011, 0.932, and 0.985. Notably, school sports see body weight, lung capacity, and standing long jump emerge as pivotal contributors, with importance values of 1.378, 1.859, and 1.491, respectively. Distinctive patterns arise in the optimization model amalgamating the random forest algorithm and the elasticity network algorithm. Angular strength, average grip strength, mean grip strength, average strength, and average strength stand out as influential factors across various sports categories. For instance, in cornering sports, average grip strength, cardiac performance index, and waist circumference exert notable influence, with importance values of 0.074, 0.073, and 0.072, respectively. In footrace and skill sports, cardiac performance index, waist circumference, and standing turn assume more profound roles, boasting importance values of 0.079, 0.08, and 0.079. Similarly, school sports exhibit the dominance of factors such as body weight, lung capacity, and standing long jump, with importance values of 0.19, 0.125, and 0.119, respectively. The amalgamation of the random forest and the elasticity network algorithms presents an even more intricate analysis of influencing factors, shedding light on the divergent health effects stemming from distinct sports categories.

### Analysis of model performance evaluation accuracy results

**Fig 8:**
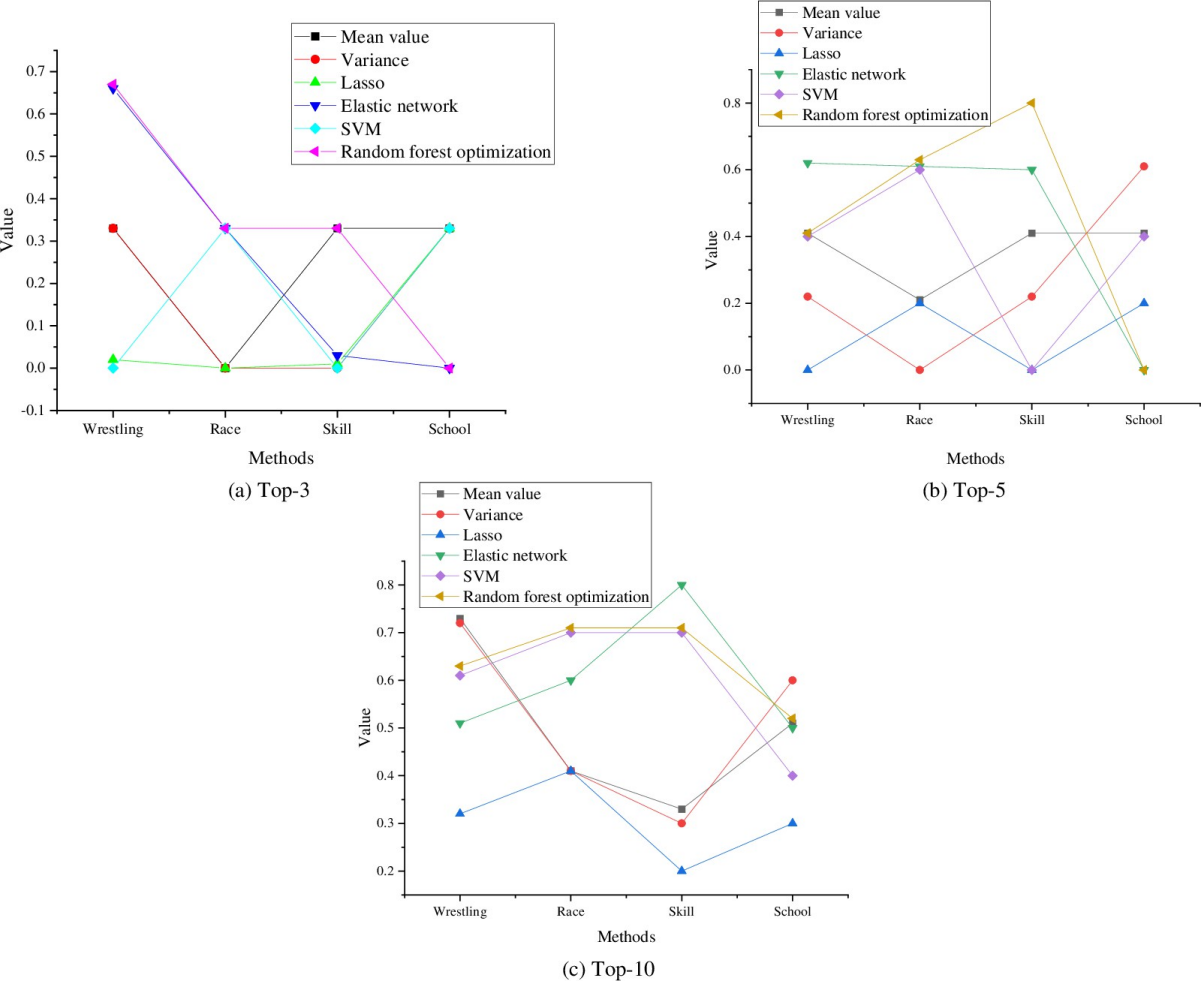
Analysis results of model effect evaluation accuracy. (a) Top-3. (b) Top-5. (c) Top-10.

A fusion model integrating the RF and Elastic Net algorithms has been devised to enhance the optimization of the sports effect evaluation model. The central focus of this study is the investigation of the impact of sports activities, with primary variables encompassing the physical condition data of young individuals and sports behavior data spanning the period from 2015 to 2020. The Top-K metric is adopted as the evaluation criterion to assess the model’s accuracy across three distinct evaluation criteria: Top-3, Top-5, and Top-10. Fig 8 illustrates the accuracy outcomes of the model’s effect evaluation across the aforementioned evaluation criteria.

As illustrated in Fig 8, the sports effectiveness evaluation model based on the elastic network algorithm yields a set of critical outcomes across varying evaluation criteria. When employing the Top-3 evaluation standard, competitive foot sports exhibit an average value of 0, while jousting sports, skill sports, and school sports consistently perform well. Upon elevating the evaluation criteria to Top-5, the mean alteration trend for each sport remains consistent, with a slight overall increase up to 0.41. Further exploration using the Top-10 evaluation criteria reveals that jousting sports boast the highest mean, followed by school sports, while skill sports exhibit a relatively lower result. It is notable that the trend of variance alteration for each sport category under the Top-3, Top-5, and Top-10 evaluation criteria aligns roughly with the mean change trend. In comparison with the traditional Lasso algorithm, the elastic network algorithm model achieves an accuracy range of 0 to 0.8 under the Top-3, Top-5, and Top-10 evaluation criteria, affirming its elevated accuracy. Conversely, the Support Vector Machine algorithmic model showcases accuracy between 0 and 0.7 across these three criteria, surpassing the traditional Lasso model and even outperforming the Elastic Network algorithmic model under the Top-10 criterion. Within the sports effect evaluation model optimized by the random forest algorithm, the accuracies of diverse sports types under the Top-3, Top-5, and Top-10 criteria consistently fall within the 0 to 0.8 range. While the range indicates marginal alteration, the overall model accuracy demonstrates enhancement compared to the support vector machine algorithm and the standalone elastic network algorithm.

## Discussion

Amidst ongoing economic progress, the emphasis on spiritual and physical well-being is growing. As a vital means of enhancing physical health, sport has gained increasing significance in people’s lives. A comprehensive and scientifically grounded understanding of sports’ role necessitates exploring sports effect assessment technologies. This study strives to achieve this goal by establishing a sports effect assessment model under the elastic network algorithm and comparing it with the conventional Lasso algorithm. Furthermore, the integration of the Random Forest algorithm with the sports effect assessment model under the elastic network algorithm is undertaken. This integration introduces the dimensions of jousting sports, footrace sports, skill sports, and school sports, thus expanding the assessment scope similar to the traditional Lasso algorithm. The accuracy of both models is assessed under three distinct evaluation criteria: Top-3, Top-5, and Top-10. This evaluation is conducted by juxtaposing the models against the Support Vector Machine algorithm. In contrast to prior studies, this study combines the elastic network algorithm and the random forest algorithm to categorize sports into the dimensions of jousting, competitive soccer, skill, and school sports. This approach facilitates the elucidation of the varied effects of diverse sports on physical health. Remarkably, the fusion model significantly enhances accuracy across all three evaluation criteria, underscoring its efficacy. Results reveal that the impacts of different sports are diverse. For instance, jousting sports primarily enhance strength, competitive foot sports positively influence bouncing ability, and skill sports bolster choice and reaction abilities, thereby expediting reaction speed. Conversely, school sports tend to amplify overall physical fitness and holistic quality. The integration of the elastic network and random forest algorithms enriches the assessment paradigm, enabling the categorization of sports effects within distinct sport-type dimensions. The fusion model exhibits high accuracy under Top-3, Top-5, and Top-10 evaluation criteria, effectively bridging a gap in sports research concerning effect assessment. Model accuracy spans from 0 to 0.8 under these three criteria, signifying its aptitude for predicting diverse sports effects. This accuracy range signifies the model’s capacity to precisely predict trends and influencing factors in sports and physical education effects, offering a dependable predictive foundation. Practically, this accuracy range signifies the model’s capability to effectively predict and categorize sports effects under varying evaluation criteria. This insight aids athletes, coaches, and health practitioners in comprehending the impacts of distinct sports on physical health, thereby guiding targeted training and adjustments for optimal health outcomes. The optimization model presented herein is poised to yield valuable insights for future youth sports selection, fostering tailored exercise strategies and advancing sports health. Ultimately, this study furnishes novel methodologies and directions for research and practical applications in the realm of sports.

## Conclusion and future directions

The evaluation of sports effects serves as a crucial measure of the efficacy of sports activities. This study aims to deepen the realm of sports data mining and enhance the technology for evaluating sports effects. In this investigation, a sports effect assessment model is developed using the elastic network algorithm, which is subsequently refined through the application of the random forest algorithm. The goal is to explore the impacts of diverse sports on physical well-being. The model’s accuracy, evaluated under the Top-K criterion, exhibits a range of 0 to 0.8, surpassing the performance of the conventional Lasso algorithm. Notably, the fusion model displays improved accuracy in assessing sports and physical activity effects across distinct dimensions while outperforming the support vector machine algorithm. The findings underscore the diverse effects of distinct sports categories on health, offering practical insights for enhancing sports health, enriching sports data mining methodologies, and charting novel directions for the sports domain. This model holds significant value in advancing sports health and elevating the standards of data mining.

Despite the achievements of this study, certain limitations are acknowledged. Primarily, the data employed lacked consideration for individual differences, potentially impacting the comprehensiveness of the outcomes. Moreover, in terms of algorithm selection, the constructed sports effect assessment model, rooted in the elastic network and random forest algorithms, predominantly embraced supervised learning methods, with less attention given to unsupervised or semi-supervised learning algorithms. For future research, including empirical studies could yield more specific youth sports data, thereby accounting for personalized variations in the dataset. Additionally, integrating diverse methodologies, such as neural networks, at the algorithmic level could enhance the model’s scientific rigor and accuracy. These enhancements have the potential to further refine the assessment of physical activity and sports effects, effectively harnessing their beneficial impact on health promotion.

## Supporting information

S1 Data (ZIP)

S1 Text (TXT)

S2 Text (TXT)
